# Feasibility of Immersive Virtual Reality for Delivering Semantic Feature Analysis to Adults with Communication Disorders and Normal Cognitive Aging

**DOI:** 10.63144/ijt.2026.6746

**Published:** 2026-06-01

**Authors:** Jennine Harvey, Megan E. Cuellar, Yihsiang Isaac Chang

**Affiliations:** 1 Communication Sciences and Disorders, Illinois State University, USA; 2 Communication Sciences and Disorders, Piedmont University, USA; 3 Technology, Illinois State University, USA

**Keywords:** Communication disorder, Normal cognitive aging, Telerehabilitation, Virtual reality

## Abstract

This study investigated the feasibility of delivering Semantic Feature Analysis (SFA) within a fully immersive virtual reality (VR) environment for adults with normal cognitive aging (NCA) and communication disorders (CSD). The scope included short-term semantic feature training, baseline assessments, and evaluation of engagement and acceptability. Thirty-five adults (11-younger, 11-older, and 13-CSD) completed a two-phase protocol involving standardized SFA and VR-delivered SFA (VRSFA) across five tasks: picture naming, associative matching, common feature identification, semantic feature questions, and integrated SFA training. Participants completed cognitive and language assessments pre- and post-intervention, with qualitative feedback gathered through thematic analysis. Quantitative data were analyzed using non-parametric tests to assess group differences and task effects. Findings indicate that immersive VR is a feasible, engaging, and promising approach for delivering evidence-based semantic-feature training for individuals with typical and impaired cognitive communication profiles. Results support continued exploration of VR as a scalable and accessible rehabilitation modality in speech-language pathology.

Prolonged life expectancy, early disease detection, and advances in medical treatment have enabled individuals to live significantly longer while managing complex and chronic medical conditions well into older adulthood. This population level demographic shift has created increased demands on healthcare systems, especially in areas related to cognitive and communication abilities, as well as age-related changes in memory, processing speed, and executive functioning, which place individuals at greater risk for functional communication decline (Blazer et al., 2015; Crimmins, 2015; Maresova et al., 2019; Thakur et al., 2013). Projections from the World Health Organization indicate that the proportion of individuals aged 60 and older will nearly double, from 12% to 22%, over the next three decades, underscoring the need to expand rehabilitation supports to meet this rapidly growing segment of the population (WHO, 2022). Additionally, best practices for aging-in-place emphasize the strategies that promote independence in activities of daily living, improve access to services, and reduce strain on healthcare and community resources (Ratnayake et al., 2022). As workforce shortages and health system costs increase, and challenge traditional service delivery, growth in digital health solutions has become essential to addressing these issues. Technology-based innovations such as virtual and remote treatment methods are emerging as promising tools to expand rehabilitative support for both typical cognitive aging and individuals living with communication disorders (Lasaponara et al., 2021; Ningrum & Kung, 2023; Tortora et al., 2024).

Adults experiencing cognitive aging may demonstrate declines in spatial ability, reasoning, prospective and episodic memory, selective and divided attention, working memory, information processing speed, and executive functioning (Blazer et al., 2015). These typical declines associated with the normal aging processes may also be exacerbated by neurological injuries or disease, resulting in acquired cognitive-communication disorders that significantly disrupt word retrieval, semantic representation, and communicative effectiveness. Examples of such disorders include aphasia, traumatic brain injury, dementia, mild cognitive impairment, and primary progressive aphasia, each of which introduces challenges for management of activities of daily living and autonomy (Bonanno et al., 2022; Calderone et al., 2023; Efstratiadou et al., 2018; Flanagan et al., 2016; Henry et al., 2019; Marshall et al., 2018; Massaro & Tompkins, 1994; Zhu et al., 2022). Individuals with these conditions often require cognitive linguistic interventions that simultaneously target language, semantic memory, working memory, and executive functioning to support communication and improve daily functioning. Given current population trends, developing versatile, effective, and scalable alternative service delivery solutions are needed to mitigate the increasing burden on rehabilitation providers and healthcare systems and ensure equitable access to person-centered care.

Virtual Reality (VR) potentially offers a novel, accessible, and cost-effective means to address these challenges by providing simulated, interactive environments that can replicate and/or approximate real-world activities and tasks essential for daily communication performance. VR platforms vary in their immersion level, sense of presence, and opportunities for interaction, ranging from non-immersive screen-based displays to fully immersive head-mounted systems equipped with motion sensors that enable naturalistic engagement with a virtual environment (Faria et al., 2023; Gunduz et al., 2023; Sokolowska, 2024). These technologies have demonstrated growing utility in neurorehabilitation, supporting both cognitive and physical training in engaging formats designed for skill generalization. Studies investigating VR-based interventions for cognitive aging have reported improved cognitive function and positive effects on memory, attention, cognitive flexibility, and executive function (Bonanno et al., 2022; Calderone et al., 2023; Rizzo et al., 2020; Tuena et al., 2019). Research has also shown that immersive virtual environments such as kitchens, supermarkets, shopping malls, and city streets provide ecologically valid contexts that mirror performance patterns observed in real-world settings, suggesting the potential to strengthen functional outcomes in communication and daily life (Allain et al., 2014; Faria et al., 2023; Kourtesis et al., 2020, 2021; Sokolowska, 2023). Beyond benefits to engagement, VR-based interventions support the transition of therapy into home settings and align well with the expansion of telepractice services, thereby enhancing access for populations with transportation, mobility, or geographic barriers (Bonanno et al., 2022; Sevcenko & Lindgren, 2022).

While the promise of VR in communication sciences and disorders is significant, using this technology therapeutically continues to require rigorous research to establish feasibility and subsequently determine implementation standards. Continued investigation is needed to define intervention intensity and duration, identify best practices for skill generalization, and develop standardized approaches for measuring clinical outcomes (Bryant et al., 2020; Rizzo et al., 2020). Research is also needed to examine cybersickness and user experience across diverse populations (Faria et al., 2023; Sokolowska, 2023; Tortora et al., 2024). Further research is also needed to establish technological design considerations such as aging and clinically friendly interfaces, familiarity in the environments selected, privacy and cybersecurity protections, and clear clinical guidelines to ensure safety and successful usability for both clinicians and patients (Bryant et al., 2020; Healy et al., 2022; Kouijzer et al., 2023; Sokolowska, 2023).

Semantic Feature Analysis (SFA) is a well-established, evidence-based intervention that targets word retrieval deficits by strengthening semantic networks through systematic activation of semantic features, thereby supporting lexical access and promoting cognitive-linguistic engagement across memory and executive function systems (Baddeley, 2003; Boyle & Coelho, 1995; Boyle, 2004, 2010; Coelho et al., 2000). Semantic Feature Analysis training has been found to improve cognitive function for persons with Aphasia, traumatic brain injury, Alzheimer’s dementia, primary progressive aphasia, and individuals experiencing declines associated with normal cognitive aging or mild cognitive impairment, indicating its broad applicability across communication-impaired populations (De Marco et al., 2021; Efstratiadou et al., 2018; Flanagan et al., 2016; Henry et al., 2019; Marshall et al., 2018; Rebstock & Wallace, 2020; Massaro & Tompkins, 1994; Taler et al., 2020). The cognitive complexity and multi-domain neural pathways associated with semantic retrieval make SFA an intervention particularly suited for delivery in an enriched, interactive virtual environment across a heterogeneous clinical demographic. Preliminary studies integrating SFA and VR suggest positive outcomes in word finding, functional communication, and skill maintenance following treatment in a simulated, non-immersive virtual world (Marshall et al., 2018). These findings support continued investigation into the use of fully immersive VR systems that may further enhance the literature regarding ecological validity, engagement, and generalization into daily communicative contexts.

Given the expanding need to provide cost-effective, accessible rehabilitation for adults with communication and cognitive decline, pairing SFA intervention with immersive VR represents a logical next step to establish feasibility and advance the literature on novel, cost-effective, and potentially scalable service delivery solutions. The purpose of this study is therefore to examine the feasibility and effects of delivering SFA in a fully immersive VR environment, compared with traditional cognitive rehabilitation therapy, for adults with normal cognitive aging and those with acquired cognitive communication disorders. In doing so, this investigation contributes to the growing literature on the evidence-based use of VR in communication sciences and disorders and aligns with calls for research on VR-based cognitive-communication outcomes that support the development of aging-friendly intervention frameworks.

## Methodology

Participants included 22 adults with normal cognitive aging and 13 with communication disorders. Groups included eleven younger adults ages 18–30 years old (Mean = 22.45, SD = 2.16), eleven older adults ages 55–90 years old (Mean = 66.91, SD = 9.68), and thirteen adults with communication disorders (CSD) ages 20–72 years old (Mean = 42.15, SD = 21.25). Gender Identity was reported as 23 women, 11 men, and 1 non-binary participant. Sex was identified as 25 females and 10 males. Individuals with communication disorders had a neurological history of their diagnosis or related etiologies verified by medical history and documentation. The communication disorders group included individuals with language or cognitive deficits. See Table 1 for Demographic and Assessment Data. Given that this was a feasibility study, a heterogeneous sample of individuals with communication disorders was included to provide insight regarding the clinical utility of this advanced technology in a typically diverse rehabilitation setting. Cognitive and language deficits result from a variety of acquired disorders and etiologies that lead to pathophysiological impairment of neurological and cognitive-communication networks. SFA has been assessed for efficacy across these populations in its traditional format. However, the implications of delivering SFA via advanced technology remain unclear, particularly factors related to cognitive load, tolerance, and outcome measures for each study participant. Baseline measures for language and cognitive-communication disorders were taken. The medical history intake worksheet was used to collect current hearing and vision evaluations. Participants who required hearing aids or visual aids (e.g., glasses or contacts) used each throughout the protocol. Furthermore, before each phase and modality of the protocol, visual and auditory acuity for the protocol stimuli was verified. All participants were proficient in the English language.

Standardized assessments for cognitive and communication functioning were used to establish baselines in general cognitive function, expressive language, phonological and auditory processing, short-term memory, working memory, cognitive processing speed, attentional control, word fluency, and word retrieval. Standardized assessments included the Montreal Cognitive Assessment (MoCA) (Nasreddine, 2010), the Boston Naming Test (BNT) (Kaplan et al., 1983), and the Woodcock-Johnson Test IV (WJ IV COG) Test of Cognitive Abilities (Schrank & Wendling, 2018). From the WCJIV Test, the following sections were administered: Section 5. Phonological Processing (Auditory Processing), Section 10. Numbers Reversed, and Section 17. Pair Cancellation. In addition, informal Word Fluency tasks were used to assess phonemic and categorical naming (dePaula et al., 2015; Gonzalez-Recober et al., 2023; Shao et al., 2014).

Test administration was approximately 30 minutes to 1 hour, depending on participant variability, with breaks available anytime. All individuals received their results regardless of study inclusion. Individuals who met age and previous diagnosis requirements were able to participate. See Table 1 for Demographic and Assessment Data. Participants were assessed by a graduate student in Communication Sciences and Disorders, who was supervised by the Principal Investigator (PI) and a certified speech-language pathologist. The PI and student research clinicians reviewed the results and questions with the participants.

Participants were recruited from a mid-west community. This primarily included posting fliers and brochures to businesses and facilities (with business and facility permission) throughout the community, such as grocery stores, restaurants, libraries, community centers, churches, independent living communities, and rehabilitation facilities. Ethical approval for this study was received from the Illinois State University Institutional Review Board (IRB-2019-568).

### Procedures

All procedures described below are consistent with the protocol description in Harvey-Northrop, Cuellar, and Chang (2023, 2026).

#### SFA Protocol Design

Semantic Feature Analysis training tasks include naming and describing category, function, action, location, association, and properties (Efstratiadou et al., 2018). To study semantic function, researchers (Taler et al., 2020) examined skills of naming, association, properties/common features, and function. The current SFA protocol integrated the skill isolation tasks reported by Taler et al. (2020) to investigate SFA skills individually and in a Semantic Feature Analysis Training Task, which includes all six SFA skills. Stimuli were evenly matched across verbs, artifacts, and biological pictures (Balota et al., 2007; Taler et al., 2020) and replicated from Taler et al. (2020). The VR SFA stimuli were type-matched for the VR kitchen environment. Due to the length of the protocol (e.g., SFA and VRSFA), data collection was modified to 10 trials per task, 50% of the Taler et al. (2020) protocol.

#### VR Protocol Design

The methods for the VR protocol incorporated guidelines from recommendations in the literature (Dermody et al., 2020; Healy et al., 2022; Rizzo et al., 2020; Tuena et al., 2020). The graphical engine to render the virtual world within the Meta Quest 2 head-mounted display (HMD) was the commercially available Unity 5.0 (“Unity,” n.d.). The virtual reality environment was designed so the user could explore the space with the standard Quest 2 controllers using only one standard button and the toggle button. To reduce the likelihood of cybersickness caused by a discrepancy between the perceived and actual body’s orientation and motion, the speed of movement and the gravity in the VR space were modified (Gallagher & Ferrè, 2018; Oh & Son, 2022). Participants were provided with a VR technology orientation session that included verification of vision, auditory, and dexterity skills; orientation to the kitchen environment; VR SFA protocol in the kitchen; and a VR environment survey. The VR environment was projected onto a SmartBoard throughout all phases of the VR protocol, allowing researchers to guide participants in the VR environment and provide feedback as needed.

### Protocol Phases

#### Phase 1- SFA Protocol

In phase 1 of the protocol, participants completed 5 SFA training tasks: Picture Naming, Associative Matching, Common Feature Identification, Semantic Feature Questions, and the Semantic Feature Analysis Task (Efstratiadou et al., 2018; Harvey-Northrop et al., 2023, 2026; Talor et al., 2020).

Task 1: Picture Naming. Picture Naming tasks are applied to examine the ability to recall the name of a given picture or object (Harvey-Northrop et al., 2023, 2026; Taler et al., 2020). Two modalities of responses, spoken and written, were trialed for picture naming (Taler et al., 2020). Participants were asked to “Name the following items” in the spoken picture naming task (Harvey-Northrop et al., 2023, 2026). Verb, artifact, and biological pictures were evenly distributed and presented individually (Balota et al., 2007; Taler et al., 2020) for ten trials. In the written picture naming task, participants were asked to “Write the name of the following items.” (Harvey-Northrop et al., 2023, 2026). Like spoken picture naming, the written picture naming task includes unique stimulus sets with an even distribution of verb, artifact, and biological pictures over ten trials. Accuracy data and error responses were collected throughout the 20 total trials of picture naming.

Task 2: Associative Matching. Associative Matching tasks are used to assess the association of two pictures, objects, or words (Harvey-Northrop et al., 2023, 2026; Taler et al., 2020). Two modalities of target stimuli, picture and typed word target, were trialed for associative matching (Taler et al., 2020). Participants were asked to “Point to the associated image” in the picture target associative matching task (Harvey-Northrop et al., 2023, 2026). The picture target was placed above a row of four equally distributed artifacts and biological pictures (Balota et al., 2007; Taler et al., 2020). Participants were required to point to the associated image in the four-picture row for ten trials. Similar to the picture target associative matching task, the typed word target associative matching task included unique stimulus target sets with an even distribution of artifacts and biological pictures (Balota et al., 2007; Taler et al., 2020), where participants were asked to “Point to the associated image” over ten trials. Accuracy data and error responses were collected throughout the 20 total trials of associative matching.

Task 3: Common Feature Identification. The Common Feature Identification tasks aim to test the capacity to recognize similar characteristics of paired objects, words, and pictures (Harvey-Northrop et al., 2023, 2026; Taler et al., 2020). In the initial common feature identification task, two words were provided verbally, and the participants were asked, “What is a common feature between those two words?” (Harvey-Northrop et al., 2023, 2026). For example, racquet and bat, where the correct response is a tool for hitting a ball during sports activities. An additional prompt, “Is there another way that these two things are similar?” was provided if the participant did not respond or if the response was vague (Taler et al., 2020, p.3). The second common feature identification task provided a written sheet of four sets of words, visually and verbally. Participants were instructed to “Choose two items. What is a common feature between those two words?” (Harvey-Northrop et al., 2023, 2026). Each subtask included ten trials where stimuli were equally distributed artifacts and biological pictures (Balota et al., 2007; Taler et al., 2020). Accuracy data and error responses were collected across the 20 total trials of common feature identification.

Task 4: Semantic Feature Questions. Semantic feature question tasks are used to examine semantic knowledge of an object, word, or picture (Harvey-Northrop et al., 2023, 2026; Taler et al., 2020). Participants were provided the following instruction during the semantic feature question task, “I am going to ask you some questions; please respond with yes or no” (Harvey-Northrop et al., 2023, 2026). For example, “Do pianos have strings?” (Taler et al., 2020, Suppl. p.6). Questions were distributed equally between biological and artifact items (Taler et al., 2020). Accuracy data and error responses were collected throughout the 12 total trials of semantic feature identification.

Task 5: Semantic Feature Analysis Training Task. The semantic feature analysis training task incorporates skills in previous tasks (naming association, feature identification, function) and includes additional skills in the areas of category, function, action, location, association, and properties (Efstratiadou et al., 2018; Harvey-Northrop et al., 2023, 2026) for examination of item description and recall. Participants were auditorily presented with six stimuli (three nouns and three verbs) and six questions for each target (Harvey-Northrop et al., 2023, 2026). Noun stimuli questions were aimed at category, function, action, location, association, and properties (Efstratiadou et al., 2018). Example questions include, “What is it used for?”, “Where do you find it?”, and “What does it look like?” (Harvey-Northrop et al., 2023, 2026). Verb stimuli questions were aimed at the subject, purpose, how, location, association, and properties (Efstratiadou et al., 2018). Example questions include, “Who usually does this?”, Why does this happen?”, and “Where does this happen?” (Harvey-Northrop et al., 2023, 2026). Accuracy data and error responses were collected throughout the 36 total trials of the semantic feature analysis training task.

#### Technology Orientation and Training

Consistent with Harvey-Northrop et al. (2023, 2026), participants were provided a 5 to 10-minute break following administration of the SFA Protocol, and the Technology Orientation and Training was then completed. The graduate research clinician provided instruction and practice to the participant on hardware, software, and the VR environment (Harvey-Northrop et al., 2023, 2026). Screening and verification of visual and auditory acuity for the VR headset and environments, and of dexterity for using the controller, were completed. Technology settings were adjusted as necessary. The VR kitchen environment was introduced, and participants were given 5 to 10 minutes to explore (Harvey-Northrop et al., 2023, 2026).

#### Phase 2- VR SFA Protocol

In the VR SFA protocol, tasks were administered in the same five areas: Picture Naming, Associative Matching, Common Feature Identification, Semantic Feature Questions, and the Semantic Feature Analysis Task (Efstratiadou et al., 2018; Harvey-Northrop et al., 2023, 2026; Talor et al., 2020) in the VR environment. The virtual environment was a kitchen, providing an immersive, ecologically valid context for engaging in intervention protocols within the activities of daily living (ADL) (Allain et al., 2014; Faria et al., 2023; Kourtesis et al., 2020, 2021; Sokolowska, 2023). For the purposes of this feasibility study, equivalent stimuli were created in the virtual environment for categories of verbs, artifacts, and biological pictures (Balota et al., 2007; Taler et al., 2020). The current VR environment does not permit in-world writing at this time. Therefore, the written subtasks were omitted from the VR SFA protocol (Harvey-Northrop et al., 2023, 2026).

Task 1: VR Picture Naming. The VR Picture Naming task included verbal picture naming (Harvey-Northrop et al., 2023, 2026). Participants were given the following instructions: “Look at the kitchen island. Name the items on the kitchen island. Now, look at the kitchen oven and counter. Name the items on the kitchen oven and counter." (Harvey-Northrop et al., 2023, 2026). Accuracy and error responses were collected across 10 total picture-naming trials in the virtual reality kitchen environment.

Task 2: VR Associative Matching. For the VR Associative Matching task, participants were provided the following sets of instructions, “Look at the kitchen island. Pick up one item. Pickup a second item in your other hand that is associated.” and “Now, complete the same task with items on the kitchen oven and counter. Pick up one item. Pick up a second item in your other hand that is associated.” (Harvey-Northrop et al., 2023, 2026). Accuracy data and error responses were collected for ten total trials of associative matching in the virtual reality kitchen environment.

Task 3: VR Common Feature Identification. In the VR Common Feature Identification task, two words were provided verbally, and the participants were asked, “Now, I will name two words. What is a common feature between those two words?” (Harvey-Northrop et al., 2023, 2026). For example, plate and frisbee, where the correct response is that they are both round disc shapes. An additional prompt, “Is there another way that these two things are similar?” was provided if the participant did not respond or if the response was vague (Harvey-Northrop et al., 2023, 2026; Taler et al., 2020, p. 3). Response accuracy and errors were collected across the ten total trials of common-feature identification in the virtual reality kitchen environment.

Task 4: VR Semantic Feature Questions. Participants were provided the following instructions for the VR Semantic Feature Questions task: “I am going to ask you some questions; please respond with yes or no” (Harvey-Northrop et al., 2023, 2026). For example, “Are earmuffs usually used in summer?” (Taler et al., 2020, Suppl. p.6). Accuracy data and error responses were collected during the 12 trials (six biological, six artifact items) (Taler et al., 2020) of semantic feature questions in the virtual reality kitchen environment.

Task 5: VR Semantic Feature Analysis Training Task. In the VR Semantic Feature Analysis task, three nouns and three verbs related to kitchen activities of daily living were verbally presented (Harvey-Northrop et al., 2023, 2026). In alignment with the SFA protocol, participants were asked the same six questions for each noun or verb stimulus. Accuracy and error responses were collected throughout the 36 total trials of the semantic feature analysis training task in the virtual reality kitchen environment.

#### Post Protocol Survey & Post Informal Word Fluency Probe

In alignment with the Harvey-Northrop et al. (2023, 2026) protocol, participants completed the VR environment survey following the VR SFA tasks. Questions about the overall experience, process, and environment were included in the survey (Harvey-Northrop et al., 2023, 2026). The SFA and VR SFA protocols both concluded with the informal word fluency probe.

#### Post-Program Participant Education

Finally, participants were provided with educational handouts regarding (1) cognitive scores and functions, (2) semantic feature analysis training and tips, and (3) virtual reality training and tips (Harvey-Northrop et al., 2023, 2026). The graduate research clinician reviewed the findings and answered any questions for the standardized assessments and recommendations for each participant under the guidance and supervision of the PI (Harvey-Northrop et al., 2023, 2026). For both the SFA and VR handouts, the graduate clinician overviewed the purpose, evidence-based research, and tips for use during everyday life, with links to resources.

#### Measures

Throughout the protocol, qualitative and quantitative data were documented (Harvey-Northrop et al., 2023, 2026). The post-protocol survey was used to gather qualitative data. Standardized pre-assessment measures, semantic function accuracy across five SFA tasks and five VR SFA tasks, and phonemic and categorical fluency probes were used to collect quantitative data.

### Analysis

Consistent with Harvey-Northrop et al. (2023, 2026), qualitative and quantitative analyses were conducted to examine the feasibility of SFA performance delivered through a VR platform compared to traditional VR administration across groups with normal cognitive aging and communication disorders.

#### Quantitative Analysis

Quantitative data were analyzed using nonparametric Mann-Whitney and Wilcoxon Signed-rank tests, with corresponding post hoc analyses.

#### Qualitative Analysis

Qualitative data were analyzed using the Qualitative Thematic Analysis (Kiger & Varpio, 2020). Data gathered from participant post-protocol surveys was examined. Researchers participated in the thematic analysis process (Kiger & Varpio, 2020), in which coding was conducted independently; each researcher identified preliminary codes and overall themes for the data. Once this stage was completed, the research team reviewed and defined themes together.

## Results

### Quantitative

#### Task Performance

Table 2 shows group mean and standard deviation across intervention performance for younger adults, older adults, and adults with communication disorders.

Non-Parametric Analyses. Due to the small sample size, group and task differences were investigated using nonparametric analyses. No significant difference in performance by age was found using the Mann-Whitney analysis, suggesting that older and younger adults performed similarly across tasks. There was a significant difference in performance between adults with normal cognitive aging compared to adults with communication disorders. The results indicate that older adults performed significantly better (M = 15.64) than adults with CSD (M = 9.85) for the SFA Task 3 – Common Feature Identification, z = −2.38, p = .017; significantly better (M = 16.91) than adults with CSD (M = 8.77) for the SFA Task 4 – Semantic Questions, z = −3.00, p=.002; and significantly better (M = 16.77) than adults with CSD (M = 8.88) for the VR SFA Task 4 – Semantic Questions, z = −2.92, p = .003. These findings suggest that individuals with communication disorders may perform similarly to individuals with normal cognitive aging using VR-enriched environments during Common Feature Identification tasks. Further, the use of VR environments involving semantic questions may not aid individuals with communication disorders in performing similarly to individuals with normal cognitive aging.

To assess if participants demonstrated a change in performance across sessions, a Wilcoxon test was performed. There was a significant difference in Task 1 – Picture Naming performance between the traditional SFA and VR sessions, z = −2.94, p = .002 (2 exact). The mean for Task 1 SFA was 94.00, while the mean for Task 1 VR SFA was 99.43, indicating that older and younger participants demonstrated increased picture naming skills in the Virtual Reality environment compared to SFA administration. Furthermore, there was a significant difference for Task 2 – Associative Matching performance between traditional SFA vs. VR session, z = −3.82, p <.001 (2 exact). The mean for Task 2 SFA was 92.00, while the mean for Task 2 VR SFA was 100, indicating that older and younger participants demonstrated increased associative matching skills in the Virtual Reality environment compared to SFA administration. Finally, there was a significant difference for Task 5 – Semantic Feature Analysis Task performance between traditional SFA vs. VR session, z = −2.40, p=.020 (2 exact). The mean for Task 5 SFA was 97.77, while the mean for Task 5 VR SFA was 99.74.

Taken together, these results suggest that there were group differences for traditional Common Feature Identification, traditional Semantic Questions, and VR Semantic Questions. Furthermore, adults with normal cognitive aging and those with communication disorders performed better during Picture Naming, Associative Matching, and Semantic Feature Analysis training tasks in the Virtual Reality environment than in the traditional SFA administration.

#### Pre-Post Probe Assessment

Non-parametric Analysis. Non-parametric analyses were performed to assess probe and age variables while accounting for sample size (Harvey-Northrop et al., 2023, 2026). The Mann-Whitney analysis results suggested no significant difference between the younger adults and older adults group’s performance, younger adults and individuals with CSD, as well as older adults and individuals with CSD across probe measures. A Wilcoxon test was performed to assess if participants exhibited a change in performance from pre/post categorical and phonemic fluency probes. Findings suggested a significant difference in categorical fluency during the post-program probe, *z* = −3.43, p <.001. The pre-program probe mean was 14.51, with the post-program probe mean of 17.66. Thus, when accounting for sample size, the earlier difference in Phonemic Probe group performance was resolved, and categorical fluency improved with the completion of the VR SFA.

#### Quantitative Results Summary

These quantitative results suggest that adults with normal cognitive aging performed comparably across the five SFA tasks and the SFA protocol delivered in the Virtual Reality environment. Older adults and adults with communication disorders perform differently across the Common Feature Analysis, Semantic Questions, and Semantic Feature Analysis Task tasks. This differential performance resolves for the Common Feature Analysis performance when in the VR environment. Significant performance improvement was observed in picture naming, associative matching, and semantic feature analysis training tasks completed in the Virtual Environment compared with the traditional therapy environment. Additionally, Categorical Fluency performance improved with the completion of the VR SFA session. While initial results are promising, research with a larger sample size is needed.

### Qualitative

A Qualitative Thematic Analysis was performed to assess the overall experience, VR environment, and protocol procedures (Harvey-Northrop et al., 2023, 2026). No group difference for the theme was found. Notably, adults with communication disorders reported the same experience as individuals with normal cognitive aging across adulthood.

#### Overall Experience

Consistent with previous findings (Harvey-Northrop et al. 2023, 2026), the major themes of the overall experience in the VR kitchen environment were that it was “fun,” “interesting,” and a “unique environment.” See Table 3 for examples of participant feedback. Modest themes were observed for potential uses of the environment, including education, training, health, and wellness. During the technological session, no participants indicated or exhibited cybersickness. In the VR SFA protocol, one participant reported that the environment was “very weird” and “uncomfortable,” referencing their eyes hurt. Per the protocol, the participant was offered the option to quit the VR environment, take a break, or sit down. None of the other participants indicated or demonstrated cybersickness during or after the VR SFA protocol. Modest themes of the environment being taxing or disorienting were observed when objects did not interact (e.g., stack) or fall (e.g., due to gravity and weight), as would typically be the case in real life. Participant feedback was used to modify item behaviors and enhance the functionality of the immersive environment.

#### Immersive Environment

As found in Harvey-Northrop, Cuellar, & Chang (2023, 2026), the major themes of environment immersion in the VR kitchen were that participants enjoyed the environment’s detail and object interactions, specifically object sounds. See Table 3 for examples of participant feedback. Furthermore, feedback indicated that a broader range of interaction opportunities, greater diversity of objects, and expanded space would be appreciated. In alignment with previous findings (Harvey-Northrop et al. 2023, 2026), there was a difference in reactions between participants with minimal to no VR experience and those familiar with or who have worked with VR platforms. Most participants reported minimal exposure to VR technology and noted the environment’s realism. Participants with more experience indicated that it was not as realistic and that greater detail was needed. Participant feedback was used when planning future development of the VR kitchen and related environments.

#### Protocol Process

All participants reported that the protocol was well-defined and easy to follow, consistent with Harvey-Northrop et al. (2023, 2026). Furthermore, participants consistently indicated that the technical session was necessary to address technology barriers. See Table 3 for examples of participant feedback. Participants offered suggestions for other uses of the environment or procedures, such as VR training in different environments and jobs, uses across age groups, student training, and health and wellness, in alignment with previous works (Harvey-Northrop et al. 2023, 2026).

#### Qualitative Results Summary

Qualitative results indicate that adults with normal cognitive aging and communication disorders report that the VRSFA protocol is a fun, interesting, and unique experience. Feedback indicates that technology training is crucial for successfully using the technology and that VR object characteristics contribute to immersive environment. Notably, participation in the VRSFA protocol prompted many participants to offer suggestions to enhance the current protocol and imagine uses for virtual reality technology and environments in general.

#### Summary Results

Quantitative and qualitative findings indicate that virtual reality technology is a feasible, interactive platform for delivering SFA interventions to individuals with NCA and CSD. Furthermore, adults with normal cognitive aging and communication disorders demonstrated comparable skills in SFA and VRSFA protocols. There was a significant increase in unprompted communication and interaction in the VR kitchen environment across groups.

## Discussion

This study examines the feasibility and impact of using SFA in a fully immersive VR environment for adults with normal cognitive aging and individuals with communication disorders. The study was also designed to investigate the preliminary clinical outcomes of VR SFA compared to traditional SFA and contribute to baseline performance data in VR clinical literature so as to inform future guidelines for implementation. Additionally, this study was designed to contribute to the literature regarding the adaptability and flexibility of delivering traditional evidence-based interventions for acquired cognitive communication impairments in fully immersive VR environments, such as the kitchen utilized in the current study.

Overall, the current study findings indicate that adults with normal cognitive aging and adults with communication disorders demonstrate comparable performance in both the traditional SFA and VR SFA tasks. More specifically, participants significantly improved in picture naming, associative matching, and semantic feature analysis training tasks in the Virtual Environment compared to the traditional therapy environment. Qualitative results support quantitative findings where all groups found the environment interesting, immersive, and unique, likely contributing to the increased communication in the VR environment. Moreover, these data may support the use of familiar VR environments for language stimulation and word recall. Furthermore, categorical fluency performance across groups improved after VRSFA completion, suggesting that SFA administered in VR increases lexical retrieval for individuals with NCA and CSD. These findings are consistent with and expand upon findings reported in Harvey-Northrop, Cuellar, and Chang (2023, 2024), indicating that SFA intervention delivered in a virtual reality kitchen environment is comparable and potentially more engaging as compared to SFA administered in a typical rehabilitation environment.

The current protocol and immersive virtual environment incorporated guidelines for SFA, VR for aging-friendly practices, as well as standards for VR implementation in healthcare and rehabilitation (Abelle et al., 2021; Healy, 2022; Khokale et al., 2023; Rizzo et al., 2020; Sevcenko & Lindgren, 2022; Taler, 2020; Tuena et al., 2020; WHO, 2021). To our knowledge, this is the first study to integrate these guidelines and recommendations into a virtual reality intervention protocol, implemented across adult NCA and CSD groups, with an evidence-based intervention. The VR CRT protocol included participant education for the communication and VR protocol and use, a protocol to reduce cybersickness and account for individual physical abilities, and software design that prioritizes patient privacy and cybersecurity-focused through the use of HIPPA and IRB-compliant Unity sandbox development (Koujzer et al., 2023; Sokolowska, 2023). Given that participants reported that the technological orientation and training sessions were critical to the protocol and the success of technology use, it seems reasonable to suggest that all future VR protocols should include a standardized technological training session to orient participants to the environment prior to clinical engagement. Additionally, virtual object characteristics (e.g., sound and visual elements) were essential to the believability of the VR immersion, which contributed to engagement and user experience satisfaction. Interestingly, participation in the VRSFA protocol prompted many participants to offer unsolicited suggestions to enhance the current protocol but also imagine uses for virtual reality technology and environments in general. These data contribute to the growing literature on VR design guidelines and suggest that incorporating end use experiences into future iterations and updates to environments is critical to increasing environment ecological validity.

In terms of the preliminary clinical outcomes obtained from the limited sessions delivered in the current study, the data suggests that participation has the potential to improve linguistic and semantic memory skills across adulthood with persons with communication disorders showing similar if not increased responsiveness. Additionally, these results demonstrate the viability of expanding SFA training to individuals with communication disorders, including individuals with acquired cognitive and language impairments (Efstratiadou et al., 2018; Marshall et al., 2018). Clinical populations that may respond well to VR interventions include mild cognitive impairment (Taler et al., 2020), and traumatic brain injury (Massaro & Tompkins, 1994) and individuals with executive function and attention deficits (Bonnanno et al., 2022; Calderone et al., 2023; Park, 2022; Zhu et al., 2022). With regard to the SFA training protocol, this study further supports the methodology of Taler et al. (2020), whereby SFA tasks were scaffolded from individual task implementation to examine semantic memory skills. This study therefore not only corroborates the feasibility of SFA in an immersive VR environment for individuals with communication disorders, but also contributes to the ever-expanding literature that supports flexible and adaptive use of traditional clinical training protocols in VR (Faria et al., 2023; Rizzo et al., 2020; Tortora et al., 2024; Yu et al., 2024)

## Limitations

While this study found similarities and differences in protocol performance for individuals with communication disorders, as previously identified (Harvey-Northrop et al., 2023, 2026), limitations are apparent that should be addressed and investigated further in future studies. First, study results and potential applications were constrained by the sample size and the number of sessions. Additionally, a heterogeneous group of individuals with CSD were included, which is representative of future end users in a rehabilitative environment but nonetheless constrains interpretation relative to specific disorders or clinical populations.

In the future, large-scale, methodologically rigorous investigations in VR for SLP interventions are needed (Dermody et al., 2020; Yu et al., 2024). Furthermore, virtual reality environments may increase executive function demands, resulting in increased cognitive load during task completion (Lawson & Mayer, 2024; Rizzo et al., 2020). These potential increases in cognitive load warrant further research and should therefore be considered in the planning and development of interventions delivered in virtual reality environments. Furthermore, spatial memory tests should be included in VR protocols to account for the dependence on spatial memory in the VR environment (Tuena, 2019). Finally, the addition of a cybersickness questionnaire, such as the VR Neuroscience questionnaire, the Simulator Sickness Questionnaire, the Virtual Reality Sickness Questionnaire, or the Cybersickness in VR Questionnaire, should be included in future studies (Faria et al., 2023; Sokolowska, 2023).

## Next Steps

Given current study findings and trends, future studies should expand protocol length, investigate a variety of evidence-based interventions, and expand population guidelines. Consistent with the current literature, future studies should examine the generalizability of skills and strategies, ethical considerations, and the effects of ecological validity. Investigation of the generalizability of skills and strategies practiced in the virtual environment to real-world activities of daily living is needed to not only determine clinical utility but also to evaluate the potential for bridging an ever-expanding gap in services available to an increasingly aging and clinical population that would benefit from rehabilitative services (Bryant et al., 2020; Son & Park, 2022; Tortora et al., 2024). Lastly, detailed guidelines are necessary to mitigate ethical concerns and potential privacy issues related to using virtual reality technology with clinical populations with communication disorders (Bryant, 2020).

## Conclusion

This study investigated the feasibility and impact of using immersive VR and SFA with adults with normal cognitive aging and individuals with communication disorders. Study results indicate that virtual reality technology is, in fact, a promising interactive platform for delivering SFA training interventions to individuals with normal cognitive aging and those with communication disorders. This study contributes to the growing examination of VR environments for SLP practice and provides a model of an adaptable, fully immersive VR environment for SLP practice, supporting the use of VR environments as a possible new service delivery platform (Harvey-Northrop et al. 2023, 2026). While preliminary findings are promising, large-scale studies are needed to standardize VR protocols across evidence-based intervention programs and to determine affordable, accessible supports for individuals with communication disorders.

## Figures and Tables

**Table 1:** Demographic and Assessment Data for Each Participant

Participant	Age	Gender	Sex	MoCA	BNT	WJ IV COG -5		WJ IV COG -10		WJ IV COG -17	

Younger Adults						Raw score	SS (68% Band)	Raw score	SS (68% Band)	Raw score	SS (68% Band)
001	22	Woman	Female	29	15	54	105 (96–113)	11	78 (72–83)	78	92 (87–97)
002	23	Man	Male	26	15	68	103 (97–108)	21	110 (106–114)	74	88 (83–94)
003	25	Woman	Female	29	13	52	93 (88–98)	23	114 (110–119)	95	104 (99–108)
004	20	Woman	Female	30	14	70	111 (103–119)	20	108 (103–112)	80	94 (89–99)
005	24	Woman	Female	30	14	50	100 (93–108	8	63 (57–69)	104	110 (105–115)
006	21	Man	Male	25	11	59	101 (93–109)	14	90 (85–95)	87	99 (94–103)
007	21	Woman	Female	26	12	64	117 (109–126)	27	125 (121–130)	99	106 (102–111)
008	22	Woman	Female	29	14	56	110 (101–118)	14	90 (85–95)	90	100 (96–105)
009	27	Woman	Female	28	15	75	113 (107–120)	28	126 (122–130)	73	87 (81–93)
010	22	Woman	Female	27	13	48	84 (75–93)	4	<40 (<40–51)	87	98 (90–107)
011	20	Woman	Female	25	10	48	88 (74–101)	2	<40 (<40–51)	66	80 (70–90)
Older Adults											
012	68	Man	Male	26	14	41	90 (86–93)	19	112 (108–116)	71	94 (105)
013	55	Man	Male	30	15	66	113 (106–119)	28	128 (124–132)	78	100 (95–105)
014	59	Woman	Female	27	15	71	121 (114–128)	25	122 (118–126)	90	111 (106–115)
015	60	Woman	Female	30	15	55	108 (101–115)	15	98 (93–102	60	84 (80–89)
016	67	Woman	Female	28	15	53	108 (101–114	17	106 (102–111)	59	88 (84–93)
017	82	Woman	Female	26	15	41	83 (78–89)	13	98 (93–103)	84	121 (117–125)
018	64	Man	Male	29	15	66	110 (105–114)	22	117 (113–121)	67	93 (88–98)
019	65	Woman	Female	29	13	50	98 (94–102)	20	113 (109–117)	91	114 (110–119)
020	73	Woman	Female	28	13	65	106 (99–114)	5	56 (41–71)	81	113 (104–123)
021	85	Woman	Female	26	15	36	87 (81–94)	5	60 (45–74)	49	95 (87–103)
022	58	Woman	Female	28	14	83	117 (108–126)	9	73 (62–84)	87	108 (99–117)
CSD											
023	22	Non-binary	Female	26	13	53	104 (95–112)	11	78 (72–83)	103	109 (104–114)
024	23	Man	Male	30	14	82	124 (117–131)	25	120 (115–124)	55	72 (67–76)
025	22	Man	Female	24	14	77	121 (113–129)	6	53 (46–60)	40	59 (54–65)
026	42	Woman	Female	27	12	51	93 (88–97)	17	100 (95–104)	107	116 (111–120)
027	64	Woman	Female	27	15	67	125 (118–132)	20	113 (109–117)	74	100 (94–105)
028	71	Man	Male	25	14	48	96 (92–99)	9	77 (72–83)	42	79 (74–84)
029	20	Woman	Female	25	13	43	89 (82–96)	12	82 (77–88)	69	83 (78–89)
030	59	Woman	Female	25	14	48	87 (81–94)	9	60 (45–74)	51	95 (87–103)
031	65	Man	Male	22	15	67	98 (91–104)	2	<40 (<40–44)	74	96 (85–107)
032	62	Man	Male	24	14	44	90 (83–97)	6	60 (47–73)	89	112 (103–120)
033	22	Woman	Female	28	12	37	71 (62–80)	6	53 (<40–66)	87	99 (90–107)
034	58	Man	Male	27	15	56	97 (90–104)	14	94 (85–104)	78	101 (90–112)
035	18	Woman	Female	22	10	31	64 (54–73)	2	<40 (<40–<40	3	<40 (<40–<40

*Note.* MoCA raw scores range 0–30. BNT raw scores range 0–15. The three WJ IV COG Subtests (5 Phonological and Auditory Processing, 10 Numbers Reversed, 17 Pair Cancellation) are presented with raw and standard scores, along with 68% confidence bands.

**Table 2:** Participant Performance across Traditional SFA and Virtual Reality SFA

	Traditional SFA			Virtual Reality SFA		

Task	Young Adults	Older Adults	CSD	Younger Adults	Older Adults	CSD
Task 1: Picture Naming	94.55 ± 6.88	95.45 ± 6.88	92.31 ± 10.13	100 ± 0	98.18 ± 6.03	100 ± 0
Task 2: Associative Matching	92.73 ± 11.04	91.82 ± 7.51	91.54 ± 9.87	100 ± 0	100 ± 0	100 ± 0
Task 3: Common Feature Identification	97.27 ± 6.47	99.09 ± 3.02	88.46 ± 13.45	94.55 ± 9.34	93.64 ± 8.09	90.77 ± 9.54
Task 4 Semantic Questions	90.91 ± 10.23	97.82 ± 3.73	87.08 ± 10.17	92.36 ± 7.19	97.73 ± 5.44	84.62± 13.64
Task 5: Semantic Feature Analysis Task	97.27 ± 4.54	99.73 ± .905	96.54 ± 6.92	99.73 ± .91	100 ± 0	99.54 ±1.13

*Note.* Accuracy is given in percentage (mean ± standard deviation).

**Table 3:** Qualitative Examples of Participant Feedback by Theme

Overall Experience	Immersive Environment	Protocol Process
“Amazing! Great to see this.”	“The detail in the VR was outstanding.”	“You explained it well"
“It was good and something new.”	“I like that you can manipulate things that are not actually there.”	"What I saw, it related to what you said.”
“I like that there is a lot of stuff in the kitchen.”	“It’s fake, but real life at the same time.”	
“I like hearing questions in the VR environment. It is more comfortable because it feels like I have more control and engaged while answering.”	“I liked the sounds, it was very realistic!”	
	“It would be cool to have someone could actually cook and practice ADLs.”	

## References

[b1-ijt-18-1-6746] Abeele V Schraepen B Huygelier H Gillebert C Gerling K Van Ee r 2021 Immersive virtual reality for older adults: Empirically grounded design guidelines ACM Transactions on Accessible Computing 14 10 1 30 10.1145/3470743

[b2-ijt-18-1-6746] Allain P Foloppe DA Besnard J Yamaguchi T Etcharry-Bouyx F Le Gall D Nolin P Richard P 2014 Detecting everyday action deficits in Alzheimer’s disease using a nonimmersive virtual reality kitchen Journal of the International Neuropsychological Society: JINS 20 5 468 477 10.1017/S1355617714000344 24785240

[b3-ijt-18-1-6746] Baddeley A 2003 Working memory and language: An overview Journal of Communication Disorders 36 3 189 208 10.1016/s0021-9924(03)00019-4 12742667

[b4-ijt-18-1-6746] Balota DA Yap MJ Cortese MJ Hutchison KA Kessler B Loftis B Neely JH Nelson DL Simpson GB Treiman R 2007 The English Lexicon Project Behavior Research Methods 39 3 445 459 10.3758/bf03193014 17958156

[b5-ijt-18-1-6746] Blazer DG Yaffe K Liverman CT Committee on the Public Health Dimensions of Cognitive Aging, Board on Health Sciences Policy, & Institute of Medicine 2015 Cognitive Aging: Progress in Understanding and Opportunities for Action National Academies Press (US) 10.17226/21693 25879131

[b6-ijt-18-1-6746] Bonanno M De Luca R De Nunzio AM Quartarone A Calabrò RS 2022 Innovative technologies in the neurorehabilitation of traumatic brain injury: A systematic review Brain Sciences 12 12 1678 10.3390/brainsci12121678 36552138 PMC9775990

[b7-ijt-18-1-6746] Boyle M Coelho CA 1995 Application of semantic feature analysis as a treatment for aphasic dysnomia American Journal of Speech-Language Pathology 4 4 94 98 10.1044/1058-0360.0404.94

[b8-ijt-18-1-6746] Boyle M 2004 Semantic feature analysis treatment for anomia in two fluent aphasia syndromes American Journal of Speech-Language Pathology 13 3 236 249 10.1044/1058-0360(2004/025) 15339233

[b9-ijt-18-1-6746] Boyle M 2010 Semantic feature analysis treatment for aphasic word retrieval impairments: What’s in a name? Topics in Stroke Rehabilitation 17 6 411 422 10.1310/tsr1706-411 21239365

[b10-ijt-18-1-6746] Bryant L Brunner M Hemsley B 2020 A review of virtual reality technologies in the field of communication disability: Implications for practice and research Disability and Rehabilitation: Assistive Technology 15 4 365 372 10.1080/17483107.2018.1549276 30638092

[b11-ijt-18-1-6746] Calderone A Carta D Cardile D Quartarone A Rifici C Calabrò RS Corallo F 2023 Use of virtual reality in patients with acquired brain injury: A systematic review Journal of Clinical Medicine 12 24 7680 10.3390/jcm12247680 38137752 PMC10743630

[b12-ijt-18-1-6746] Coelho CA McHugh R Boyle M 2000 Semantic feature analysis as a treatment for aphasic dysnomia: A replication Aphasiology 14 133 142 10.1080/026870300401513

[b13-ijt-18-1-6746] Crimmins EM 2015 Lifespan and healthspan: Past, present, and promise The Gerontologist 55 6 901 911 10.1093/geront/gnv130 26561272 PMC4861644

[b14-ijt-18-1-6746] De Marco M Blackburn DJ Venneri A 2021 Serial recall order and semantic features of category fluency words to study semantic memory in normal ageing Frontiers in Aging Neuroscience 13 678588 10.3389/fnagi.2021.678588 34413764 PMC8370562

[b15-ijt-18-1-6746] de Paula JJ Paiva GCC Costa DS 2015 Use of a modified version of the switching verbal fluency test for the assessment of cognitive flexibility Dementia & Neuropsychologia 9 3 258 264 10.1590/1980-57642015DN93000008 29213970 PMC5619367

[b16-ijt-18-1-6746] Dermody G Whitehead L Wilson G Glass C 2020 The role of virtual reality in improving health outcomes for community-dwelling older adults: Systematic review Journal of Medical Internet Research 22 6 e17331 10.2196/17331 32478662 PMC7296414

[b17-ijt-18-1-6746] Efstratiadou EA Papathanasiou I Holland R Archonti A Hilari K 2018 A systematic review of semantic feature analysis therapy studies for aphasia Journal of Speech, Language, and Hearing Research 61 5 1261 1278 10.1044/2018_JSLHR-L-16-0330 29710193

[b18-ijt-18-1-6746] Faria AL Latorre J Silva Cameirão M Bermúdez I Badia S Llorens R 2023 Ecologically valid virtual reality-based technologies for assessment and rehabilitation of acquired brain injury: a systematic review Frontiers in Psychology 14 1233346 10.3389/fpsyg.2023.1233346 37711328 PMC10497882

[b19-ijt-18-1-6746] Flanagan KJ Copland DA van Hees S Byrne GJ Angwin AJ 2016 Semantic Feature Training for the treatment of anomia in Alzheimer Disease: A preliminary investigation Cognitive and Behavioral Neurology: Official Journal of the Society for Behavioral and Cognitive Neurology 29 1 32 43 10.1097/WNN.0000000000000088 27008248

[b20-ijt-18-1-6746] Gallagher M Ferrè ER 2018 Cybersickness: A multisensory integration perspective Multisensory Research 31 7 645 674 10.1163/22134808-20181293 31264611

[b21-ijt-18-1-6746] Gonzalez-Recober C Nevler N Shellikeri S Cousins KAQ Rhodes E Liberman M Grossman M Irwin D Cho S 2023 Comparison of category and letter fluency tasks through automated analysis Frontiers in Psychology 14 1212793 10.3389/fpsyg.2023.1212793 37901072 PMC10600440

[b22-ijt-18-1-6746] Gunduz ME Bucak B Keser Z 2023 Advances in stroke neurorehabilitation Journal of Clinical Medicine 12 21 6734 10.3390/jcm12216734 37959200 PMC10650295

[b23-ijt-18-1-6746] Harvey-Northrop J Cuellar M Chang I 2023 October 30–November 2 Impact of delivering semantic feature analysis in traditional vs. virtual reality environments [Conference presentation abstract] One hundredth annual convention of the American Congress of Rehabilitation Medicine Atlanta, GA, United States

[b24-ijt-18-1-6746] Harvey-Northrop J Cuellar M Chang I 2026 Virtual reality & normal cognitive aging: Implications for semantic feature analysis training. [Manuscript submitted for publication] Department of Communication Disorders, Illinois State University

[b25-ijt-18-1-6746] Healy D Flynn A Conlan O McSharry J Walsh J 2022 Older adults’ experiences and perceptions of immersive virtual reality: Systematic review and thematic synthesis JMIR Serious Games 10 4 e35802 10.2196/35802 36472894 PMC9768659

[b26-ijt-18-1-6746] Henry ML Hubbard HI Grasso SM Dial HR Beeson PM Miller BL Gorno-Tempini ML 2019 Treatment for word retrieval in semantic and logopenic variants of primary progressive aphasia: Immediate and long-term outcomes Journal of Speech, Language, and Hearing Research 62 8 2723 2749 10.1044/2018_JSLHR-L-18-0144 PMC6802912 31390290

[b27-ijt-18-1-6746] Kaplan EF Goodglass H Weintraub S 1983 The Boston Naming Test 2nd Edition Lea & Febiger Philadelphia

[b28-ijt-18-1-6746] Khokale R S Mathew G Ahmed S Maheen S Fawad M Bandaru P Zerin A Nazir Z Khawaja I Sharif I Abdin ZU Akbar A 2023 Virtual and augmented reality in post-stroke rehabilitation: A narrative review Cureus 15 4 e37559 10.7759/cureus.37559 37193429 PMC10183111

[b29-ijt-18-1-6746] Kiger ME Varpio L 2020 Thematic analysis of qualitative data: AMEE Guide No. 131 Medical Teacher 42 8 846 854 10.1080/0142159X.2020.1755030 32356468

[b30-ijt-18-1-6746] Kouijzer MMTE Kip H Bouman YHA Kelders SM 2023 Implementation of virtual reality in healthcare: A scoping review on the implementation process of virtual reality in various healthcare settings Implementation Science Communications 4 1 67 10.1186/s43058-023-00442-2 37328858 PMC10276472

[b31-ijt-18-1-6746] Kourtesis P Collina S Doumas LAA MacPherson SE 2021 Validation of the virtual reality everyday assessment lab (VR-EAL): An immersive virtual reality neuropsychological battery with enhanced ecological validity Journal of the International Neuropsychological Society 27 2 181 196 10.1017/S1355617720000764 32772948

[b32-ijt-18-1-6746] Kourtesis Panagiotis Korre Danai Collina Simona Doumas Leonidas MacPherson Sarah 2020 Guidelines for the development of immersive virtual reality software for cognitive neuroscience and neuropsychology: The development of Virtual Reality Everyday Assessment Lab (VR-EAL), a neuropsychological test battery in immersive virtual reality Frontiers in Computer Science 1 12 10.48550/arXiv.2101.08166

[b33-ijt-18-1-6746] Lasaponara S Marson F Doricchi F Cavallo M 2021 A scoping review of cognitive training in neurodegenerative diseases via computerized and virtual reality tools: What we know so far Brain Sciences 11 5 528 10.3390/brainsci11050528 33919244 PMC8143131

[b34-ijt-18-1-6746] Lawson A Mayer R 2024 Individual differences in executive function affect learning with immersive virtual reality Journal of Computer Assisted Learning 40 3 1068 1082 10.1111/jcal.12925

[b35-ijt-18-1-6746] Maresova P Javanmardi E Barakovic S Barakovic Husic J Tomsone S Krejcar O Kuca K 2019 Consequences of chronic diseases and other limitations associated with old age - a scoping review BMC Public Health 19 1 1431 10.1186/s12889-019-7762-5 31675997 PMC6823935

[b36-ijt-18-1-6746] Marshall J Devane N Edmonds L Talbot R Wilson S Woolf C Zwart N 2018 Delivering word retrieval therapies for people with aphasia in a virtual communication environment Aphasiology 32 9 1054 1074 10.1080/02687038.2018.1488237

[b37-ijt-18-1-6746] Massaro M Tompkins C 1994 Feature analysis for treatment of communication disorders in traumatically brain-injured patients: An efficacy study Clinical Aphasiology 22 245 256

[b38-ijt-18-1-6746] Nasreddine ZS Phillips NA Bédirian V Charbonneau S Whitehead V Collin I Cummings JL Chertkow H 2005 Montreal Cognitive Assessment (MoCA) [Database record] APA PsycTests https://mocacognition.com/ 10.1111/j.1532-5415.2005.53221.x 15817019

[b39-ijt-18-1-6746] Ningrum DNA Kung WM 2023 Challenges and perspectives of neurological disorders Brain Sciences 13 4 676 10.3390/brainsci13040676 37190641 PMC10136594

[b40-ijt-18-1-6746] Oh H Son W 2022 Cybersickness and its severity arising from virtual reality content: A comprehensive study Sensors 22 4 1314 10.3390/s22041314 35214216 PMC8963115

[b41-ijt-18-1-6746] Park J-H 2022 Effects of virtual reality-based spatial cognitive training on hippocampal function of older adults with mild cognitive impairment International Psychogeriatrics 34 2 157 163 10.1017/S1041610220001131 32616109

[b42-ijt-18-1-6746] Ratnayake M Lukas S Brathwaite S Neave J Henry H LPCMH, ATR, NCC;1, BS-c;5 2022 Aging in place: Are we prepared? Delaware Journal of Public Health 8 3 28 31 10.32481/djph.2022.08.007 PMC9495472 36177171

[b43-ijt-18-1-6746] Rebstock AM Wallace SE 2020 Effects of a combined semantic feature analysis and multimodal treatment for primary progressive aphasia: Pilot study Communication Disorders Quarterly 41 2 71 85 10.1177/1525740118794399

[b44-ijt-18-1-6746] Rizzo A Gambino G Sardo P Rizzo V 2020 Being in the past and perform the future in a virtual world: VR applications to assess and enhance episodic and prospective memory in normal and pathological aging Frontiers in Human Neuroscience 14 297 10.3389/fnhum.2020.00297 32848672 PMC7417675

[b45-ijt-18-1-6746] Schrank FA Wendling BJ 2018 The Woodcock–Johnson IV: Tests of cognitive abilities, tests of oral language, tests of achievement Flanagan DP McDonough EM Contemporary intellectual assessment: Theories, tests, and issues 4th ed 383 451 The Guilford Press

[b46-ijt-18-1-6746] Sevcenko K Lindgren I 2022 The effects of virtual reality training in stroke and Parkinson’s disease rehabilitation: A systematic review and a perspective on usability European review of aging and physical activity: Official journal of the European Group for Research into Elderly and Physical Activity 19 1 4 10.1186/s11556-022-00283-3 35078401 PMC8903585

[b47-ijt-18-1-6746] Shao Z Janse E Visser K Meyer AS 2014 What do verbal fluency tasks measure? Predictors of verbal fluency performance in older adults Frontiers in Psychology 5 772 10.3389/fpsyg.2014.00772 25101034 PMC4106453

[b48-ijt-18-1-6746] Sokołowska B 2023 Impact of virtual reality cognitive and motor exercises on brain health International Journal of Environmental Research and Public Health 20 5 4150 10.3390/ijerph20054150 36901160 PMC10002333

[b49-ijt-18-1-6746] Sokołowska B 2024 Being in virtual reality and its influence on brain health-an overview of benefits, limitations and prospects Brain Sciences 14 1 72 10.3390/brainsci14010072 38248287 PMC10813118

[b50-ijt-18-1-6746] Son C Park JH 2022 Ecological effects of VR-based cognitive training on ADL and IADL in MCI and AD patients: A Systematic review and meta-analysis International Journal of Environmental Research and Public Health 19 23 15875 10.3390/ijerph192315875 36497946 PMC9736197

[b51-ijt-18-1-6746] Taler V Monetta L Sheppard C Ohman A 2020 Semantic function in mild cognitive impairment Frontiers in Psychology 10 3041 10.3389/fpsyg.2019.03041 32038404 PMC6987430

[b52-ijt-18-1-6746] Thakur R Banerjee A Nikumb V 2013 Health problems among the elderly: a cross-sectional study Annals of Medical and Health Sciences Research 3 1 19 25 10.4103/2141-9248.109466 23634324 PMC3634218

[b53-ijt-18-1-6746] Tortora C Di Crosta A La Malva P Prete G Ceccato I Mammarella N Di Domenico A Palumbo R 2024 Virtual reality and cognitive rehabilitation for older adults with mild cognitive impairment: A systematic review Ageing Research Reviews 93 102146 10.1016/j.arr.2023.102146 38036103

[b54-ijt-18-1-6746] Tuena C Serino S Dutriaux L Riva G Piolino P 2019 Virtual enactment effect on memory in young and aged populations: A systematic review Journal of Clinical Medicine 8 5 620 10.3390/jcm8050620 31067784 PMC6572276

[b55-ijt-18-1-6746] Tuena C Pedroli E Trimarchi PD Gallucci A Chiappini M Goulene K Gaggioli A Riva G Lattanzio F Giunco F Stramba-Badiale M 2020 Usability issues of clinical and research applications of virtual reality in older people: A systematic review Frontiers in Human Neuroscience 14 93 10.3389/fnhum.2020.00093 32322194 PMC7156831

[b56-ijt-18-1-6746] World Health Organization 2021 April Age-friendly in practice Age-friendly world Geneva, Switzerland https://extranet.who.int/agefriendlyworld/age-friendly-practices/

[b57-ijt-18-1-6746] World Health Organization 2022 October Aging and health World Health Organization https://www.who.int/news-room/factsheets/detail/ageing-and-health

[b58-ijt-18-1-6746] Yu J Wu J Liu B Zheng K Ren Z 2024 Efficacy of virtual reality technology interventions for cognitive and mental outcomes in older people with cognitive disorders: An umbrella review comprising meta-analyses of randomized controlled trials Ageing Research Reviews 94 102179 10.1016/j.arr.2023.102179 38163517

[b59-ijt-18-1-6746] Zhu K Zhang Q He B Huang M Lin R Li H 2022 Immersive virtual reality-based cognitive intervention for the improvement of cognitive function, depression, and perceived stress in older adults with mild cognitive impairment and mild dementia: Pilot pre-post study JMIR Serious Games 10 1 e32117 10.2196/32117 35188466 PMC8902670

